# Life Seeds and Disinfection

**Published:** 1890-02-01

**Authors:** 


### The Hospital

A Weekly Journal of

Science, Medicine, Nursing, and Philanthropy.

*For Hospitals, Asylums, Medical Practitioners, Students, Nurses, Families, Parishes, Congregations, and the Charitable Public.*

Vol. VII. No. 175.] [February 1, 1890.

### Life Seeds and Disinfection.

Mr. Wynter Blyth is not quite satisfied with the names which modern scientists have given to the small organisms whose presence in the human body under certain circumstances gives rise to disease. These small particles are known by the common appellations of "germs," "microbes," "bacteria," and the like. Mr. Blyth thinks that a simpler name would be "small life seeds"; and if the general spread of true knowledge available for everyday use be one of the objects of science, Mr. Blyth is undoubtedly quite right. There may be, there is, a certain scientific convenience in the use of scientific terms derived from the Greek and Latin languages. But if every scientist were half as great a man as he thinks he is he would take care always to use, not only the accepted scientific terms in his writings, but also their equivalents in the mother tongue. We have no notion whatever of a man's being worthy of the name of "man," much less of that of "great man," who wishes to confine his knowledge to the circle of the learned. However able and successful his "special" work may be, he himself is a very poor creature.

Mr. Blyth wishes to teach us all to make our houses, hospitals, and other public buildings healthy, and to keep them so. The Hospitals Association have published in pamphlet form a short paper read by him before their body a week or two ago. The pamphlet is No. 39 of the Hospitals Association pamphlets. It is eminently scientific and eminently simple. Those who wish to know all about the reason of disinfection, the methods of disinfection, and the proof of its reality and success had better get Mr. Wynter Blyth's eight-page pamphlet.

The ancients used to practise disinfection. But they neither knew why they practised it, nor the best methods of carrying it out, nor to what extent they were successful or failed in their operations. We know all about these points. The very school boy knows for example that the ordinary air is full of living germs, or, as Mr. Wynter Blyth would call them, "small life seeds." Some of these, so far as we know, are always and everywhere harmless; others are capable of producing diseases more or less various in their character and more or less deadly in their operation. We disinfect because we wish to destroy these disease-producing life seeds.

Not only have we a science of the development and behaviour of "life seeds," but we have also a well understood and scientific art of compassing their destruction. Mr. Wynter Blyth speaks of what he calls "mechanical" disinfection. This is neither more nor less than the application of the art of scrubbing and scouring thoroughly. Take, for example, the ward of a hospital which has been inhabited by men suffering from infectious disease. Clear out beds, chairs, tables, curtains, blinds, and every other article of furniture whatsoever. What have you left? The walls, the ceiling, the floor, the windows, the ledges, and shelves, and the space contained within. A great variety of life seeds must be either settled down at rest on walls, floor, and ceiling, or they must be floating about in the air. If, now, abundance of soap, hot water, floorcloths, window-cloths, limewash, brushes, and energetic men and women be brought in, it is clear that the life seeds that are settled down on floor, or ceiling, or wall will be washed by myriads into the slop-pail and sent packing, half or entirely drowned, into the congenial habitat of the common sewer. It might seem that in this method the air of the ward is left entirely untouched; but, as a matter of fact, the steam from the hot water and the unusual bustle have probably caused most of the floating life seeds to "settle" on walls or ledges, and thus the air has been at least to a large extent purified as well as the walls and ceilings. When, by way of conclusion, all the windows are thrown wide open for at least a week, it is probable that that particular ward will be in a certain degree purified. That is the old method of disinfecting hospitals and other public and private buildings, and when carried out with perfect thoroughness is not without its merits.

Another method of disinfection is by the use of heat. Heat is a very powerful disinfectant, much more so than cold. Very few germs or life seeds seem to die of cold. It is probable that most of them have completely escaped the influenza epidemic. Yellow fever yields a germ which it is said can be destroyed by intense cold—below freezing point. But so far as is known, other germs are quite capable of surviving the intensest degree of cold that is likely to be applied to them. On the other hand, water raised to no higher a temperature than the ordinary boiling point is quite sufficient to kill and destroy every kind of disease germ known to science. One of the commonest forms of life seed experimented upon by bacteriologists is the germ of the disease called "anthrax." The anthrax germ exists in two forms, as do probably most disease germs. These are known as the "strong" and the "tender" state, and it may be taken for granted that they answer to the mature and the immature condition. Spore-anthrax will live for a certain time even when treated with a 25 per cent. solution of carbolic acid. Yet the boiling of these same germs for a single minute is sufficient to kill them all. Heat, but especially moist or steam heat, is thus found to be an excellent disinfectant. Dry heat, on the other hand, does not produce anything like the same results.

The third and last method of disinfection considered by Mr. Wynter Blyth is the "chemical. In our times, and in domestic circles, this is the commonest of all. How do we know when we have used a disinfectant that we have disinfected anything? If the smell be destroyed the great probability is that disinfection has been effectual. But scientific men have much more convincing methods of proof than the mere destruction of smell. They take, for example, a mouse, and dipping the point of a lancet into an anthrax solution exactly as they might dip it into a drop of vaccine lymph, they scratch the skin of the mouse with the poisoned point of the lancet. That mouse will die of anthrax in forty-eight hours. If now the same solution of anthrax be thoroughly disinfected, a dozen mice may be scratched with a dozen dipped lancets and not one of them will take any harm. Experimenting in this way it is easy to prove not only what substances are actual disinfectants, but also their disinfecting potency. It is found that there is a very great difference in the efficacy of disinfectants. Some, though truly classed as disinfecting substances, are exceedingly feeble and slow in their action; others, again, destroy with the utmost rapidity and energy every form of germ life they come in contact with. It is important to bear in mind, as a matter of practical moment, that all disinfectants are poisonous. Any substances, says Mr. Wynter Blyth, that are advertised as non-poisonous are not disinfectants, although they may be deodorants.

It has been our endeavour to make quite clear to every reader who is capable of reasoning at all that the modern practice of disinfection is founded in well-ascertained scientific facts, and that those facts may be quite easily comprehended and valued by every person of ordinary intelligence. The aim of the disinfector is to secure everywhere perfect cleanliness and perfect atmospheric purity. Every disinfectant may be regarded as a more or less potent atmospheric soap. Combining, therefore, the most perfect cleanliness with the free use of potent disinfectants, we create for ourselves an atmosphere and general surroundings free from disease germs. That that is a gain of incalculable value, no reasonable man will venture to deny.

